# Nrf2, NF-κB and PPARβ/δ mRNA Expression Profile in Patients with Coronary Artery Disease

**DOI:** 10.5935/abc.20190125

**Published:** 2019-12

**Authors:** Jaqueline Ermida Barbosa, Milena Barcza Stockler-Pinto, Beatriz Oliveira da Cruz, Ana Carla Tavares da Silva, Juliana Saraiva Anjos, Claudio Tinoco Mesquita, Denise Mafra, Ludmila F. M. F. Cardozo

**Affiliations:** Universidade Federal Fluminense - Programa de Pós-Graduação em Ciências Cardiovasculares, Niterói, RJ - Brazil

**Keywords:** Coronary Artery Disease, Oxidative Stress, Inflammation, Obesity, Hypertension, Dyslipidemias, Risk Factors/prevalence, Myocardial Infarction, Heart Failure

## Abstract

**Background:**

Oxidative stress and inflammation are present in coronary artery disease (CAD) and are linked to the activation of the transcription nuclear factor kappa B (NF-κB). To attenuate these complications, transcription factors like nuclear factor erythroid 2-related factor 2 (Nrf2) and peroxisome proliferator-activated receptor-β/δ (PPARβ/δ) can be activated to inhibit NF-κB. However, the available data on expression of NF-κB, Nrf2 and PPARβ/δ in CAD patients are limited.

**Objective:**

To evaluate the expression of the transcription factors NF-κB and Nrf2 and PPAR𝛽/𝛿 in CAD patients.

**Methods:**

Thirty-five patients (17 men, mean age 62.4 ? 7.55 years) with CAD and twelve patients (5 men, mean age 63.50 ? 11.46 years) without CAD were enrolled. Peripheral blood mononuclear cells (PBMCs) were isolated and processed for mRNA expression of Nrf2, NF-κB, NADPH: quinone oxidoreductase 1 (NQO1) and PPARβ/δ mRNAs using quantitative real-time polymerase chain reaction (qPCR). p < 0.05 was considered statistically significant.

**Results:**

There was no difference in the mRNA expressions of Nrf2 (1.35 ? 0.57), NF-κB (1.08 ? 0.50) or in the antioxidant enzyme NQO1 (1.05 ? 0.88) in the CAD group compared to the group without CAD (1.16 ? 0.76, 0.95 ? 0.33, 0.81 ? 0.55, respectively). However, PPARβ/δ was highest expressed in the CAD group (1.17 ? 0.86 vs. 0.56 ? 0.34, p = 0.008).

**Conclusion:**

The main finding of this study was the PPARβ/δ being more expressed in the PBMC of patients with CAD compared to the control group, whereas no differences were observed in Nrf2 or NF-κB mRNA expressions.

## Introduction

Of all cardiovascular diseases (CVD), coronary artery disease (CAD) is the leading cause of death and high expenditure on medical assistance in the world, and is typically a chronic disease with progression over years or decades.^1-3^ CAD, also known as coronary arteriosclerotic heart disease or coronary heart disease, is characterized by narrowing of the arteries in the heart that supply blood, oxygen, and nutrients to the cardiac tissue.^4^

Although there has been a steady decline in the incidence of CVD in recent years, the prevalence of CVD risk factors (hypertension, high cholesterol and obesity) has been increasing. Smoking, obesity, high blood pressure (BP), high total cholesterol and low-density lipoprotein, low high-density lipoprotein, diabetes and advanced age are the main risk factors for CVD^5,6^ and are directly related to endothelial dysfunction with low bioavailability of nitric oxide, causing vasoconstriction, oxidative stress and inflammation.^7,8^ Oxidative stress is present in both etiology and progression of myocardial infarction, congestive heart failure, atherosclerosis and hypertension.^9^

Oxidative stress arises when there is an imbalance between the reactive oxygen species (ROS) production and the capacity of the antioxidant defense systems of the body,^10^ while inflammation is a biological response to oxidative stress where the cell starts producing proteins, enzymes and other compounds to restore homeostasis.^11^ Oxidative stress is responsible for inflammation by several mechanisms, one of which is the direct activation of the nuclear transcription factor kappa B (NF-κB) by the ROS. NF-κB regulates the transcription of several genes encoding proinflammatory cytokines, chemokines and adhesion molecules of leukocytes.

In this direction, it is important to evaluate factors that attenuate both inflammation and oxidative stress. Nuclear factor erythroid 2-related to factor 2 (Nrf2) has been associated with cytoprotective effects and its accumulation leads to an increase in the transcription of antioxidant response elements (ARE)-regulated genes encoding antioxidant and phase 2 detoxifying enzymes and can be considered a protective factor against both oxidative stress and inflammation.^12-14^ Under basal conditions, Nrf2 is inactive in the cytoplasm and is inhibited by its cytosolic repressor protein, Kelch-like ECH-associated protein 1 (Keap1), which through the action of certain substances, including the ROS, which alters the conformation, decouples Nrf2 and thereby facilitates accumulation and nuclear translocation of Nrf2. In the nucleus, Nrf2 binds to regulatory sequences called AREs acting on genes that encode antioxidant and phase II detoxifying enzymes, including NADPH: quinone oxidoreductase 1 (NQO1).^15^ The role of Nrf2 in reducing inflammation is related to the ability to antagonize NF-κB indirectly by removing ROS. In addition, antioxidant enzymes appear to act directly on the reduction of inflammatory mediators.^15^ Besides Nrf2, another target that has attracted interest and attention from research is the peroxisome proliferator-activated receptor-β/δ (PPARβ/δ). However, the biological functions of PPARβ/δ and its effectiveness as a therapeutic target in the treatment of hypertension and CVD have not been elucidated.^16^ PPARβ/δ is the predominant subtype in the heart and several lines of evidence suggest a cardioprotective function of PPARβ/δ.^17^ Preclinical studies suggest that PPARβ/δ activation promotes antihypertensive effects in established animal models^18^ and the pharmacological activation of PPARβ/δ prevents endothelial dysfunction and downregulates inflammatory responses.^19,20^ Furthermore, PPARβ/δ suppresses the activities of several transcription factors, including the NF-κB.^21^ Based on the fact that there are no studies about gene expression of Nrf2, NF-κB and PPARβ/δ and its profile in CAD patients, the objective of this study was to evaluate the transcription factors NF-κB and Nrf2 and PPARβ/δ mRNA expression in patients with CAD.

## Methods

### Subjects

Forty-seven patients were enrolled in this study through a convenience sample where patients composed each group according to the presence or absence of CAD. Thirty-five patients (17 men and 18 women, mean age 62.4 ± 7.5 years, BMI 28.9 ± 4.9 kg/m^2^) with CAD and/or abnormal findings of myocardial perfusion scintigraphy comprised the CAD group and twelve patients (5 men and 7 women, mean age 63.5 ± 11.5 years, 26.5 ± 6.2 kg/m^2^) without CAD comprised the group without CAD. Eligible patients were older than 18 and attended the Nuclear Medicine Section at Hospital Universitário Antônio Pedro (Niterói, Rio de Janeiro, Brazil) to undergo myocardial scintigraphy. Patients with infection, cancer, chronic kidney disease (estimated glomerular filtration rate <60 mL/min), acquired immune deficiency syndrome (AIDS) and autoimmune disease were excluded. The control group consisted of hypertensive, dyslipidemic and/or diabetic patients not diagnosed with CAD, from the same hospital.

### Anthropometric Measures

Anthropometric measurements were made by a trained staff member using standard techniques. Body mass index was calculated as weight in kilograms divided by height in square meters.^22^

### Blood pressure assessment

BP was measured by the indirect method using auscultatory technique with sphygmomanometer and appropriate cuff in accordance with the dimensions of the patient's arm. Aneroid arterial pressure device - AD-2 was used on caster (pedestal), brand UNITEC Hospitalar (INMETRO ML 095 2007/ANVISA 10432300016). To assess BP, the procedure was initially explained to the patient who was resting for more than five minutes. The patient was sitting, feet resting on the floor, back resting on the chair, arm at heart level (mid-point of the sternum), supported, free of clothing, with the palm of the hand facing upwards and the elbow slightly flexed. HA was defined when systolic BP (SBP) values were greater or equal to 140 mmHg.^23^

### Analytic procedures and sample processing

Blood was collected from each participant in the morning, after 12-hour overnight fasting, using a tube containing EDTA anticoagulant (1.0 mg/mL). Plasma was centrifugated and separated (15 min, 3000×*g*, 4°C) and stored at −80°C until analysis.

Peripheral blood mononuclear cells (PBMCs) were collected, blood samples with EDTA were diluted in PBS and cells were separated in 5 mL Histopaque (Sigma-Aldrich) by centrifugation at 1800 g for 30 min. PBMCs were collected and washed twice with cold PBS and re-suspended and stored (−80°C) with 1 mL of Recovery^TM^ cell culture freezing medium (Thermo Fisher Scientific) for RNA isolation.

### Biochemical and inflammation parameters

Total cholesterol, LDL-cholesterol, HDL-cholesterol, triglyceride, glucose and ultra-sensitive C-reactive protein levels were determined using Bioclin® automatic biochemical analyzer kits (Bioclin BS-120 Chemistry Analyzer). LDL-c was calculated using the Friedewald et al. equation.^24^

### Real-time quantitative PCR analysis

Nrf2, NF-κB, NQO1 and PPARβ/δ mRNA expressions were evaluated using real-time quantitative PCR (qPCR) from PBMCs according to Cardozo et al.^25^ TaqMan^®^ Gene Expression Assays (Applied Biosystems) were used to detect Nrf2 (Hs00975961_g1), NF-κB (Hs00765730_m1), NQO1 (Hs00168547_m1), PPARβ/δ (Hs00975961_g1) mRNA and the control gene GAPDH (Hs02758991_g1).

### Statistical analysis

Shapiro-Wilk test was applied to test sample distribution. Results were expressed as mean ± SD (age, BMI, SBP, lipidic profile, glucose, Nrf2, NF-B, NQO1, PPARβ/δ), median (interquartile range) (CRP) or percentage (hypertension, dyslipidemia, diabetes), as applicable. Unpaired Student’s *t*-test was used to compare the variables and groups with normal distribution and the Mann-Whitney-Wilcoxon test was used for nonparametric data. Correlations between variables were assessed by Pearson’s or Spearman coefficient correlation according to the distribution of the sample. A significance level of 5% was accepted. Statistical analyses were performed using the SPSS 19.0 software package (Chicago, IL, USA).

## Results

In the CAD group, 82.8% presented abnormalities on myocardial perfusion scintigraphy (65.5% myocardial ischemia, 27.6% myocardial fibrosis, and 6.9% fibrosis and myocardial ischemia). Regarding the duration of disease, 71.4% were diagnosed with CAD from 1 to 5 years, 17.1% from 6 to 10 years and 11.5% from 10 to 15 years. According to the clinical history of patients with CAD, 54.2% performed some type of procedure before the study: 8.7% cardiac catheterization, 34.3% percutaneous transluminal coronary angioplasty, 5.7% percutaneous transluminal coronary angioplasty and cardiac catheterization and 5.7% percutaneous transluminal coronary angioplasty and coronary artery bypass grafting. Moreover, 62.8% of the CAD patients and 30.8% of the control group were smokers. Considering the use of medication, in the CAD group, 68.5% used β-adrenergic blockers, 17.4% angiotensin-converting enzyme inhibitor, 77.1% statins, 28.5% calcium channel blockers, 51.4% diuretic, 37.2% nitrate, 54.3% acetyl salicylic acid, 62.8% losartan potassium, 34.8% oral hypoglycemic agents and 11.43% insulin. In the control group, 53.8% used β-adrenergic blocker, 15.4% angiotensin-converting enzyme inhibitor, 46.2% statins, 30.8% calcium channel blocker, 53.8% diuretic, 7.7% nitrate, 61.5% acetyl salicylic acid, 69.2% losartan potassium, 38.5% oral hypoglycemic agents and 7.7% insulin. No statistical differences were found between groups related to the use of medication or smoking.

Clinical profile and biochemical parameters are shown in Table 1. Also, the CAD group presented lower total cholesterol, LDL-cholesterol and HDL-cholesterol compared to the group without CAD (Table 1).

**Table 1:** Clinical profile and biochemical of the patients of the study

Parameters	Group without CAD (n = 12)	CAD Group (n = 35)	p value
Men/women (n)	5/7	17/18	0.99
Age (years)	63.5 ± 11.5	62.4 ± 7.5	0.70
Hypertension (%)	91.7	97.1	0.81
Dyslipidemia (%)	75	74.2	0.67
Diabetes (%)	16.7	37.1	0.84
BMI (kg/m^2^)	26.5 ± 6.2	28.9 ± 4.9	0.17
SBP (mmhg)	137.5 ± 23.0	138.0 ± 18.6	0.69
DBP (mmhg)	82.5 ± 9.6	82.8 ± 8.2	0.90
Total cholesterol (mg/dL)	200 ± 59.4	163.3 ± 46.7	0.03
LDL-cholesterol (mg/dL)	109.3 ± 53.3	79.9 ± 33.3	0.03
HDL-cholesterol (mg/dL)	65.1 ± 21.3	45.3 ± 9.9	0.002
Triglyceride (mg/dL)	128.2 ± 57.3	130.6 ± 71.8	0.79
Glucose (mg/dL)	115.2 ± 44.6	103.7 ± 36.4	0.13
CRP (mg/L)	0.6 (0.4-4.0)	2.0 (0.12-8.7)	0.25

CAD: coronary artery disease; BMI: body mass index; SBP: systolic blood pressure; DBP: diastolic blood pressure CRP: C-reactive protein. Parametric data expressed as mean±SD and nonparametric data expressed with median, 15^th^ and 75^th^ quartiles.

No differences were found in the transcription factors Nrf2 and NF-κB or in the NQO1 mRNA expression comparing the CAD group with the group without CAD. In contrast, the PPARβ/δ was more expressed in the CAD group (Table 2). We considered that the inclusion of diabetic patients did not interfere with the results. No correlations were found.

**Table 2:** mRNA expression levels in the group without CAD and the CAD Group

Parameters	Group without CAD	CAD Group	p value
Nrf2	1.16 ± 0.76	1.35 ± 0.57	0.35
NF-B	0.95 ± 0.33	1.08 ± 0.50	0.58
NQO1	0.81 ± 0.55	1.05 ± 0.88	0.37
PPARβ/δ	0.56 ± 0.34	1.17 ± 0.86	0.008

Nrf2, NF-kB, NOQ1 and PPAR β/δ mRNA expression was performed in PBMC by real-time quantitative PCR. Data were expressed as mean ± SD. CAD: coronary artery disease.

## Discussion

Studies have evaluated systemic inflammation through PBMC gene expression.^26,27^ The importance of studying PBMCs as a strategy to evaluate targets of inflammation-related metabolic pathways to explore CVD for a better understanding of the architecture of these diseases was emphasized. The hypothesis would be that the PBMCs could reflect inflammatory mechanisms in a more specific way compared to serum/plasma.^28^ Thus, the present study investigates the transcription factors NF-κB and Nrf2 and PPARβ/δ mRNA expression in PBMCs of CAD patients. CVD patients are usually exposed to inflammation and oxidative stress. Nrf2 protects the body against these conditions because it is related to the synthesis of antioxidant enzymes and is capable of antagonizing NF-κB involved in inflammatory induction.

Several studies have shown that NF-κB plays an important role in the development of CVD.^29-31^ It was demonstrated that ischemia rapidly induced NF-κB activation in the myocardium of rats.^29^ Wilson et al.^30^ showed that NF-κB was increased in the coronary atheromatous plaque in humans and its expression was predominantly associated with macrophages, foam cells and vascular smooth muscle cells. In addition, its expression was increased in acute coronary syndromes and associated with the intercellular adhesion molecule 1 (ICAM-1).^30^ NF-κB inhibition in endothelial cells resulted in reduced development of atherosclerosis and was correlated with reduced expression of pro-inflammatory cytokines, chemokines and adhesion molecules in the aortas of mice fed with cholesterol-rich diet.^31^

Some studies demonstrate that, as a protection mechanism, in an early stage of diseases, Nrf2 has its activity increased in order to avoid damage induced by ROS. In the final stage, due to the chronicity and/or severity of the disease, this protection mechanism may become saturated by the excess of EROs leading to the reduction of Nrf2^32,33^ or the Nrf2 appears to be insufficiently capable of antagonizing NF-κB and it remains high.^26^

Despite this, the effects of CAD on the Nrf2-Keap1 system are not well established. However, patients with CAD had lower gene expression of Nrf2/ARE and glutathione (GSH).^27^

An important phase of atherosclerotic plaque formation is endothelial infiltration well established by macrophages and formation of foam cells. In rats, Nrf2 is an important component in this process, as macrophages exposed to oxidized LDL promoted increased expression of Nrf2, which indirectly protected macrophages from oxi-LDL mediated lesions through phase II antioxidant enzymes.^34^ In addition, the absence of Nrf2 in macrophages from mice consuming a high-fat diet increased the formation of foam cells and the progression of atherosclerosis, suggesting that Nrf2 is important in resistance to atherosclerosis.^35^ Increased expression of Nrf2 at this stage of development of atherosclerosis is important because the effects on heme oxygenase-1 (HO-1) expression, which produces antiatherogenic effects as a reduction in the formation of foam cells^36^ and NQO1 also proved to be important in the protection against atherosclerosis.^37^

In the present study, there were no differences in the Nrf2 or NF-κB mRNA expression between patients in the CAD group and the group without CAD possibly due to the fact that the patients in the two groups were elderly, hypertensive and/or diabetic, demonstrating that both groups did not include healthy patients. In addition, all the patients used several medications with potential antioxidant effect.^38,39^ With age, expression of several Nrf2 downstream targets declined.^40^ It is still important to emphasize that both hypertension and diabetes are related to increased oxidative stress, accumulation of reactive oxygen species and inflammation.^9,41^

In the present study, the PPARβ/δ was high compared to the patients without CAD. It seems to be protective since it has been shown that the adequate balance of PPARβ/δ activation in the different cardiac cell types may be important for potential cardioprotective effects of PPARβ/δ.^42^ An*in vivo* study showed that cardiac specific overexpression of PPARβ/δ led to increased myocardial glucose utilization and did not alter cardiac function but exerted a protective effect on ischemia/reperfusion-induced myocardial injury.^43^ In addition, cardiac PPARβ/δ deletion in mice resulted in cardiac dysfunction, hypertrophy and congestive heart failure.^17^ Additionally, PPARβ/δ has been described in several biological functions, including cell survival.^44,45^ Studies show that inflammation, ROS and oxidized LDLs induce endothelial cell apoptosis, representing the beginning of the development of atherosclerotic lesions.^45^ Thus, assays performed on keratinocytes have shown that increased production of proinflammatory cytokines is capable of elevating PPARβ/δ expression, which in turn regulates the expression of apoptosis-related genes, resulting in increased resistance to cell death.^44^

Given the importance of PPARβ/δ and the transcription factors NF-κB and Nrf2 effects for the CAD patients - the Nrf2 orchestrating the production of antioxidant and phase 2 detoxifying enzymes being considered a protective factor against both oxidative stress and inflammation,^46^ PPARβ/δ promoting cardioprotection^42^ and NF-κB regulating inflammation^12^ - a better understanding of how they are expressed in CAD patients is useful so that strategies can be used in an attempt to modulate these transcription factors. Some studies proposed that nutrients containing plant-based Nrf2 inducers may help to improve the Nrf2-Keap1 system.^25,47^

This study presented a range of limitations that warrant consideration. Firstly, this study should have a healthy control group for comparison. Secondly, it would be interesting to stratify the results by risk factor and scintigraphy results, but the sample was not large enough for this. Thirdly, unfortunately, we did not perform another Nrf2, NF-κB and PPARβ/δ target genes that encode antioxidant enzymes and proinflammatory cytokines to confirm the Nrf2, NF-κB and PPARβ/δ expression network. Furthermore, it was not possible to calculate non-HDL cholesterol. Further studies should be encouraged to explore this issue. Considering these limitations, this was a very well-controlled protocol, which allowed us to conclude that the results are considerably relevant.

## Conclusion

The present study revealed increased expression of PPARβ/δ in the PBMC of CAD patients while no differences were observed in Nrf2 or NF-κB mRNA expressions. These findings may lead to possible therapies, targets and future research for treatment in these patients.
